# Emerging synthetic biology-assisted technologies for overcoming antibiotic resistance: CRISPR-Cas, bacteriophage, microbiome, and metabolic engineering-based solutions

**DOI:** 10.71150/jm.2512002

**Published:** 2026-03-31

**Authors:** Yujeong Oh, Hyunjin Lee, Sungho Jang

**Affiliations:** 1 Department of Bioengineering and Nano-Bioengineering, Incheon National University, Incheon 22012, Republic of Korea; 2 Research Center for Bio Materials & Process Development, Incheon National University, Incheon 22012, Republic of Korea; 3 Division of Bioengineering, College of Life Sciences and Bioengineering, Incheon National University, Incheon 22012, Republic of Korea

**Keywords:** antibiotic resistance, synthetic biology, CRISPR-Cas, bacteriophage engineering, microbiome engineering, metabolic engineering

## Abstract

Antibiotic resistance has become a critical global health challenge due to the decreased efficacy of existing antibiotics and the emergence of multidrug-resistant pathogens. In particular, the rapid horizontal transfer of resistance genes and the diverse mechanisms by which bacteria acquire resistance have significantly undermined the effectiveness of conventional therapeutic strategies, revealing fundamental limitations in current infectious disease management. In this context, synthetic biology provides a promising framework to overcome the limitations of conventional antibiotics by integrating engineering principles with bioengineering approaches, thereby enabling precise and programmable control of biological processes. These synthetic biology-based approaches offer substantial potential for developing sustainable and highly specific antimicrobial strategies. This review comprehensively examines recent advances in synthetic biology-assisted antimicrobial strategies, including CRISPR-Cas systems, bacteriophage engineering, microbiome engineering, and metabolic engineering-driven antibiotic discovery. Collectively, these approaches represent a precision antimicrobial paradigm that enables selective targeting of resistant bacteria while preserving microbiome homeostasis. These strategies also provide new directions for limiting resistance dissemination and guiding the development of next-generation therapeutics.

## Introduction

The discovery of penicillin in 1928 revolutionized the treatment of infectious diseases and significantly improved global health outcomes. However, the widespread and often indiscriminate use of antibiotics has accelerated the rise of antimicrobial resistance (AMR), now recognized as a major global health crises. AMR contributes to increased treatment failure and mortality (Llor and Bjerrum, 2014), and the World Health Organization (WHO) warns that deaths from drug-resistant infections may exceed those from cancer by 2050 (Dehbanipour and Ghalavand, 2022). Antibiotic resistance has intensified globally due to the combined effects of bacterial adaptation, horizontal gene transfer, and limited innovation in antibiotic development. Under antibiotic selection pressure, bacteria employ multiple defense strategies. Drug-inactivating enzymes such as β-lactamases, target modification exemplified by rifampicin resistance through *rpoB* mutations, and activation of efflux pumps that reduce intracellular drug accumulation collectively contribute to resistance phenotypes (Mao et al., 2025; Munita and Arias, 2016). Resistance traits also disseminate rapidly across microbial populations via horizontal gene transfer, often mediated by plasmids and other mobile genetic elements; the global spread of the carbapenemases among Enterobacteriaceae highlights the accelerating impact of these mechanisms (Ochman et al., 2000; Tacconelli et al., 2018).

Compounding these challenges, the discovery and development of new antibiotic classes has slowed significantly, largely because high R&D costs, regulatory hurdles, and limited commercial returns have reduced industry investment, thereby limiting available treatment options for infections caused by multidrug-resistant pathogens (Årdal et al., 2020). Collectively, these factors suggest that alternative antimicrobial strategies are increasingly necessary.

As AMR continues to diminish the effectiveness of conventional antibiotics, alternative therapeutic strategies have gained increasing attention, with synthetic biology emerging as a major driver of innovation. Synthetic biology applies rational engineering principles to biological systems, enabling the programmable modification of genetic circuits and microbial functions (Perrino et al., 2021). These capabilities help address key limitations of conventional antibiotics, including low target specificity and the rapid emergence of resistance. Among these approaches, CRISPR-Cas systems provide a sequence-specific platform for selectively cleaving or suppressing resistance genes (Pursey et al., 2018). Engineered bacteriophages further expand targeting precision by enabling host-range modification and improving pathogen specificity (Kilcher and Loessner, 2019). In parallel, advances in microbiome and metabolic engineering support the development of engineered live biotherapeutics and facilitate the discovery of novel antimicrobial compounds (Mimee et al., 2018). Together, these innovations position synthetic biology as a promising framework for next-generation antimicrobial development.

**Fig. 1.**
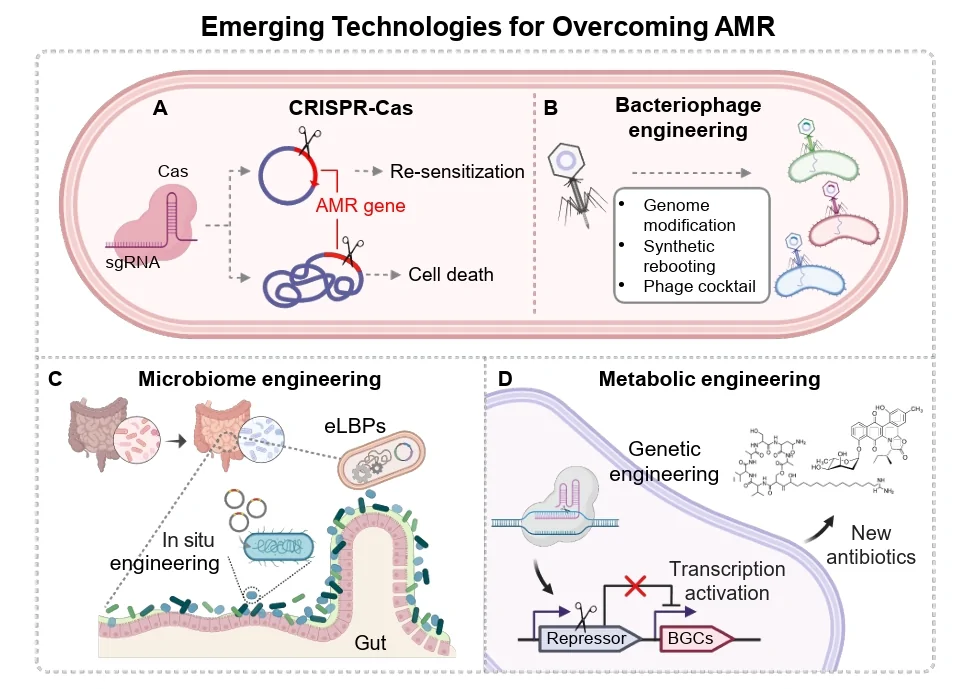
Emerging technologies for overcoming antimicrobial resistance (AMR). Overview of four major technological approaches under development to overcome AMR. (A) The CRISPR–Cas (Clustered Regularly Interspaced Short Palindromic Repeats) systems target resistance genes on chromosomes or plasmids, inducing bacterial cell death or restoring antibiotic susceptibility. (B) Synthetic biology–based bacteriophage engineering improves host-specific killing of resistant bacteria through genome modification, synthetic rebooting, and phage cocktail design. (C) Microbiome engineering modulates gut microbial ecosystems and limits the spread of antibiotic resistance genes using engineered live biotherapeutic products (eLBPs) and in situ genetic manipulation. (D) Metabolic engineering activates silent biosynthetic pathways and controls cellular metabolism to enable the production of next-generation antimicrobial compounds.

This review examines how these synthetic biology-assisted approaches, in conjunction with existing therapeutic strategies, are reshaping the landscape of antimicrobial development. We highlight recent progress in CRISPR-Cas-mediated resistance control, engineered bacteriophages, microbiome engineering, and synthetic biology-driven antibiotic discovery, emphasizing how these emerging strategies can address key limitations of conventional antibiotics and contribute to improved AMR management (Fig. 1).

## CRISPR-Cas System

The CRISPR-Cas (Clustered Regularly Interspaced Short Palindromic Repeats) system enables the selective recognition and cleavage of antibiotic resistance genes within bacterial cells. This capability directly addresses critical limitations of conventional antibiotics, including nonspecific killing and resistance development (Greene, 2018). When integrated with synthetic biology, CRISPR-Cas systems can be engineered into modular and environmentally responsive regulatory platforms with multiplex control capacity, allowing dynamic modulation of antimicrobial functions (Frusteri Chiacchiera et al., 2025).

### CRISPR-Cas–based strategies against AMR

**Fig. 2.**
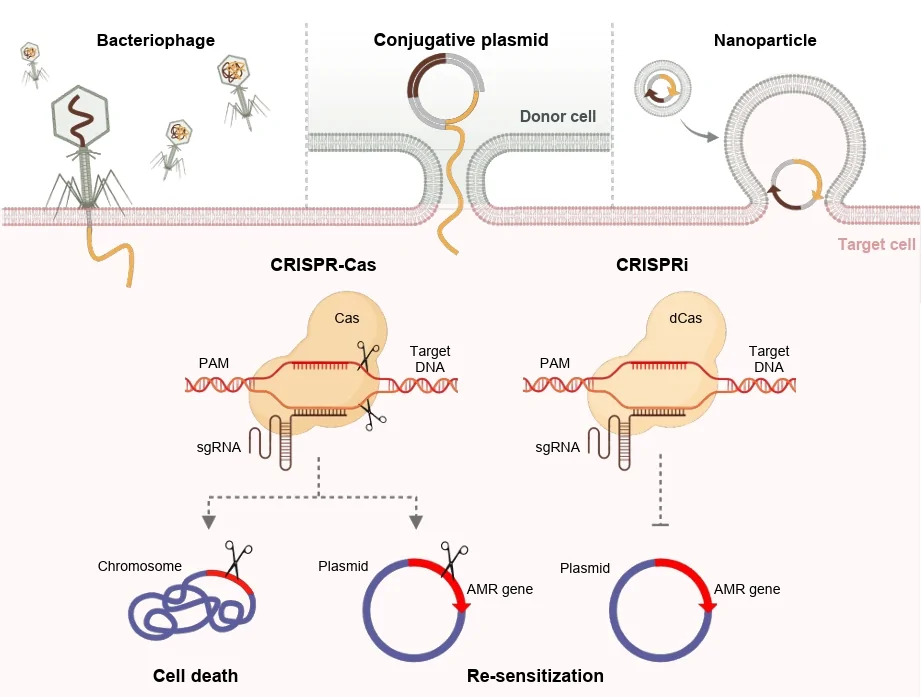
Antimicrobial activity of the CRISPR-Cas systems. Illustration of the major antimicrobial strategies mediated by the CRISPR-Cas system. Active Cas nucleases cleave bacterial chromosomes or plasmids carrying antibiotic resistance genes, leading to cell death of resistant pathogen or restoration of antibiotic susceptibility. When using catalytically inactive Cas9 (dCas9), transcription of resistance genes is repressed, resulting in re-sensitization without genome cleavage. CRISPR-Cas components can be delivered to target bacteria via bacteriophages, conjugative plasmids, or nanoparticle-based systems.

The CRISPR-Cas systems mediate antimicrobial activity primarily by inducing double-strand breaks (DSBs) in chromosomal or plasmid-encoded resistance determinants, thereby restoring antibiotic susceptibility (Mayorga-Ramos et al., 2023) (Fig. 2). Cas9, Cas12f1, and Cas3 have been shown to completely eliminate plasmid-encoded carbapenem resistance genes, resulting in full resensitization to antibiotics. Each Cas protein exhibits distinct mechanistic advantages, such as progressive DNA degradation by Cas3 and enhanced delivery efficiency associated with the smaller size of Cas12f1, highlighting complementary therapeutic potential among CRISPR platforms (Huang et al., 2025). In parallel, a probiotic *Escherichia coli* strain engineered with CRISPR-based targeting of eight clinically relevant resistance genes was protected against the acquisition of resistance determinants, significantly reducing transformation- and phage-mediated uptake as well as suppressing conjugation-mediated plasmid transfer into the engineered strain (Lee et al., 2025).

Beyond nuclease-mediated DNA cleavage, CRISPR interference (CRISPRi) employs a catalytically inactive Cas9 (dCas9) to repress gene expression by blocking transcription initiation or elongation. This approach has been successfully applied to silence resistance genes, virulence factors, and biofilm-associated determinants (Li et al., 2020; Yao et al., 2022). More recently, CRISPRi delivered via a conjugative plasmid was shown to restore antibiotic susceptibility in multidrug-resistant *E. coli* and impair bacterial growth (Frusteri Chiacchiera et al., 2025). Collectively, both nuclease-based CRISPR systems and CRISPRi provide complementary strategies for the eradication or suppression of AMR determinants.

While CRISPR-Cas and CRISPRi systems have demonstrated strong potential for overcoming AMR, their therapeutic application critically depends on efficient delivery into target bacteria. Accordingly, several delivery strategies have been explored, including bacteriophage-based, conjugative plasmid-based, and nanoparticle-based platforms (Fig. 2). Bacteriophages enable host-specific delivery but are limited by narrow host range, conjugative plasmids allow population-level spread with limited specificity, and nanoparticle-based systems offer a non-viral alternative but raise concerns regarding cytotoxicity, immunogenicity, and in vivo safety (Lin et al., 2017; Neil et al., 2021; Wei et al., 2020).

### Limitations of CRISPR-Cas–based antimicrobial strategies

Despite the strong therapeutic potential of CRISPR-Cas antimicrobials, several challenges remain regarding delivery efficiency, bacterial escape mechanisms, and biosafety. Phage-, conjugative plasmid-, and nanoparticle-mediated delivery platforms each exhibit inherent limitations. Narrow host specificity, strain-dependent transfer efficiency, and cytotoxic or immunogenic effects represent the major constraints of these delivery approaches, respectively (Kim et al., 2024a; Neil et al., 2020; Wei et al., 2020). In addition, bacteria can evade CRISPR targeting through mutations in protospacer adjacent motifs (PAMs) or spacer sequences, or by expressing anti-CRISPR proteins such as AcrIIA4, which directly inhibit Cas9 activity (Marino et al., 2020). Therefore, although CRISPR-Cas tools offer programmable and selective control of resistant pathogens (Greene, 2018), significant advances are still required to ensure efficient delivery, durability against bacterial countermeasures, and in vivo safety prior to clinical application (Kim et al., 2024a; Neil et al., 2020; Wei et al., 2020).

## Engineering of Bacteriophages

Bacteriophages are viruses that infect bacteria with high host specificity due to receptor-mediated recognition and controlled genome delivery (Lin et al., 2017; Rakhuba et al., 2010). Their specificity, self-amplification, low toxicity, and ability to degrade biofilms have renewed interest in phage therapy as an alternative strategy against multidrug-resistant pathogens (Abedon, 2015; Barbu et al., 2016). However, clinical application remains limited by challenges such as narrow host range, inconsistent infection efficiency, and difficulties in isolating suitable phages. Synthetic biology-based phage engineering provides tools to address these challenges by enabling genome modification or full-genome reconstruction.

### Bacteriophage engineering approaches

Targeted phage genome editing has been widely employed to enhance host range and infection efficiency. Using the bacteriophage recombineering of electroporated DNA (BRED) platform, the temperate phage Milagro was converted into a lytic form, resulting in a substantial expansion of its host range across multiple *Burkholderia* species (Yao et al., 2023). In parallel, modular replacement of tail fiber genes in the genetically well-characterized P2 phage enabled simultaneous binding to diverse enteric pathogens. Incorporation of a *cas9* payload further conferred Cas9-mediated bactericidal activity upon infection (Fa-Arun et al., 2023). Conversely, continuous evolution strategies have been used to fine-tune phage-host interactions, selectively enhancing infection efficiency toward specific hosts, as demonstrated for bacteriophage T7 (Holtzman et al., 2020). Such approaches are particularly relevant for precision phage therapy and microbiome modulation, where selective targeting is essential.

Beyond targeted editing, phage genome synthesis-or synthetic rebooting-enables complete reconstruction of phage genomes. Using ΦX174 as a template, generative artificial intelligence (AI) was applied to design 16 synthetic genomes that exhibited markedly altered genetic architectures while retaining infectivity, faster replication kinetics, and potent bactericidal activity against *E. coli* (King et al., 2025). Furthermore, integrated platforms combining in vitro genome design, synthesis, and screening using cell-free extracts have been established, providing a rapid and programmable route for generating customized synthetic bacteriophages (Levrier et al., 2024). More recently, plasmid-dependent phages have emerged as precision tools for directly targeting the drivers of AMR dissemination. Phage PDP46 selectively utilizes conjugative pili as its receptor, thereby eliminating only bacteria harboring resistance plasmids and demonstrating potential as a selective modulator of gut microbial communities (Jung et al., 2025).

Synthetic biology–driven phage engineering has further expanded beyond single-phage modification toward rational cocktail design that maximizes synergy with antibiotics. Phage cocktails composed of genetically diverse members effectively suppressed the growth of *Pseudomonas aeruginosa* and *Staphylococcus aureus*. In addition, systematic blueprinting of phage–antibiotic interactions has been proposed, thereby providing a conceptual framework for combination therapies aimed at suppressing AMR (Kim et al., 2024a).

### Limitations of synthetic biology-based bacteriophage engineering

Although bacteriophage therapy has re-emerged as a promising alternative to conventional antibiotics, several barriers continue to hinder its clinical translation. Bacteria can readily acquire phage resistance through receptor modification or activation of endogenous defense systems, including CRISPR-Cas–mediated immunity (Elois et al., 2023; Labrie et al., 2010). In addition, administered phages may be rapidly cleared by the host immune system, raising concerns regarding therapeutic stability and unintended inflammatory responses (Elois et al., 2023). Within the complex and heterogeneous environment of the human gut, bacteriophage performance can be further compromised by diverse environmental variables, including microbial community structure, spatial heterogeneity, and physicochemical conditions (Jung et al., 2025). Addressing these challenges will require not only advanced genetic engineering approaches but also a deeper mechanistic understanding of phage–host–immune system interactions.

## Microbiome Engineering

The gut microbiome is a major reservoir of antibiotic resistance genes and supports their intra- and interspecies transfer (Merrick et al., 2023). Conventional antibiotics disrupt this ecosystem by eliminating both pathogens and beneficial commensals, increasing dysbiosis, resistant overgrowth, and horizontal gene transfer (Fishbein et al., 2023; Robinson and Young, 2010). Synthetic biology-based microbiome engineering offers a targeted solution by deploying genetically programmed microbes or engineered circuits capable of operating in the gut environment. This approach aims to suppress dissemination of antibiotic resistance genes and restore microbial balance, providing a foundation for precise and sustainable antimicrobial strategies (Charbonneau et al., 2020).

### Microbiome engineering approaches

Engineered live biotherapeutic products (eLBPs) can be designed to suppress antibiotic-resistant pathogens by producing antimicrobial molecules or competitively reshaping microbial communities. A *Lactococcus lactis*-based eLBP was engineered to secrete a split, heterodimeric β-lactamase that degrades β-lactam antibiotics locally in the gut, thereby minimizing antibiotic-induced dysbiosis, limiting enrichment of antibiotic resistance genes, and preserving colonization resistance while maintaining systemic antibiotic exposure (Cubillos-Ruiz et al., 2022). Another strategy employs a modular bacteriocin expression platform built on non-pathogenic *E. coli* strains capable of expressing and secreting multiple bacteriocins; as a proof of concept, the system produced and secreted enterocin A and B and exhibited strong in vitro antimicrobial activity against *Enterococcus faecalis* and vancomycin-resistant *Enterococcus faecium* (Rutter et al., 2024).

Beyond introducing engineered microbes such as eLBPs, recent study has explored in situ genetic modification of native gut microbiota to reduce dissemination of antibiotic resistance genes. One approach involves engineering an *E. coli* Nissle 1917 probiotic strain with a conjugative plasmid (TP114) carrying a CRISPR-Cas9 killing module programmed to cleave AMR genes in target bacteria (Neil et al., 2021). In a mouse gut microbiota model, a single administration eliminated over 99.9% of target antibiotic-resistant *E. coli*. Furthermore, in a *Citrobacter rodentium* infection model, four consecutive days of treatment achieved therapeutic outcomes comparable to conventional antibiotic therapy. More recent work demonstrated species- and site-specific genome editing within complex microbial communities by combining environmental transformation sequencing (ET-seq), which identifies genetically tractable members, with DNA-editing all-in-one RNA-guided CRISPR–Cas transposase (DART) systems that mediate targeted DNA insertion without isolating the host organisms, achieving high on-target specificity in soil and infant gut microbiota (Rubin et al., 2022). Collectively, these studies highlight in situ microbiome engineering as a promising framework for reprogramming microbial ecosystems and mitigating AMR at the community level.

### Limitations of synthetic biology-based microbiome engineering

Synthetic biology-based microbiome engineering offers ecosystem-level control of AMR but still faces key limitations. eLBP strategies raise biosafety concerns, including potential horizontal gene transfer, uncontrolled persistence, and difficulty regulating engineered functions in vivo (Charbonneau et al., 2020). Likewise, DNA-based in situ gene delivery approaches can, in principle, modulate microbiomes with high specificity, but current implementations still face substantial challenges related to host range, delivery and ecological predictability in complex communities (Sheth et al., 2016). Despite these challenges, ongoing advances in containment design, delivery optimization, and dynamic control systems position microbiome engineering as a promising next-generation precision therapeutic approach for combating AMR.

## Metabolic Engineering and Synthetic Biology for Novel Antibiotic Development

Synthetic biology offers a framework to address antibiotic resistance by enabling rational design of antimicrobial molecules rather than relying on naturally discovered compounds. Through genetic circuit engineering and metabolic pathway reprogramming, it is possible to construct novel antimicrobial agents and improve productivity and structural diversity beyond conventional antibiotics. This approach enables next-generation therapeutics that can strategically target resistant pathogens.

Synthetic biology-based strategies enable the activation of silent biosynthetic gene clusters through precise manipulation of regulatory elements and gene circuitry. In *Streptomyces cyanogenus* S136, plasmid-based overexpression of a heterologous AdpA successfully activated the otherwise silent lcm biosynthetic gene cluster, leading to the production of lucensomycin, a polyene antibiotic with potent antifungal activity (Yushchuk et al., 2021). In *Streptomyces venezuelae*, CRISPRi-mediated knockdown of the cluster repressor JadR2 or CRISPRa-mediated activation of the *jadJ-V* operon each triggered jadomycin B production, demonstrating the utility of CRISPR-based rewiring of endogenous regulatory networks (Ameruoso et al., 2022). In *Paenibacillus brasilensis*, a cytosine base editor was used to inactivate four of five biosynthetic gene clusters, enabling genetic dereplication and allowing the remaining cluster to be characterized, which led to the discovery of a previously uncharacterized antimicrobial compound, bracidin (Kim et al., 2024b). The resulting compound exhibited antimicrobial activity against Gram-positive bacteria as well as antifungal effects.

Beyond native hosts, synthetic biology has also expanded antibiotic discovery by reconstructing entire biosynthetic pathways in model microorganisms. Model organisms such as *E. coli* and *Saccharomyces cerevisiae* are increasingly used as synthetic biology platforms for heterologous expression of complex antibiotic biosynthetic pathways (Yook et al., 2025). In *E. coli*, the salivabactin polyketide-nonribosomal peptide pathway was reconstructed on two medium-copy plasmids. Through systematic strain and pathway optimization, the authors improved metabolic flux and alleviated production-associated cytotoxicity, ultimately achieving a 22-fold increase in salivabactin titer compared to the native producer; LC-MS and NMR analyses confirmed that the heterologously produced compound was identical to the native salivabactin (Ko et al., 2025). Similary, two type II polyketide synthase pathways were reconstituted in *E. coli* by exploiting native transcriptional coupling between dimeric ketosynthase subunits, yielding soluble ketosynthase expression and functional polyketide production (Liu et al., 2020).

Structure-guided molecular engineering and computational design are emerging as powerful strategies for creating next-generation antibiotics capable of overcoming classical resistance mechanisms. Cresomycin, a conformationally constrained, bridged macrobicyclic oxepanoprolinamide antibiotic, was engineered through preorganized molecular design to maintain high-affinity ribosomal binding even in the presence of rRNA methylation, resulting in markedly improved MIC values and reduced bacterial burden against multidrug-resistant pathogens (Wu et al., 2024). Generative AI platforms have enabled de novo antibiotic discovery. Using in silico screening of large chemical libraries, haliacin-a compound structurally distinct from existing antibiotics-was identified and shown to exhibit bactericidal activity against *Mycobacterium tuberculosis* and carbapenem-resistant Enterobacteriaceae (Stokes et al., 2020). In addition, newly designed molecules such as NG1 and DN1 demonstrated strong in vivo efficacy, including potent activity against multidrug-resistant *Neisseria gonorrhoeae*. Notably, DN1 also exhibited activity against methicillin-resistant *S. aureus* (MRSA) (Krishnan et al., 2025). Collectively, these approaches highlight the accelerating potential of structure- and computation-guided design in expanding the antimicrobial arsenal.

### Limitations of metabolic engineering and synthetic biology-driven antibiotic discovery

Despite progress in synthetic biology-driven antibiotic discovery, several technical barriers persist. In Actinomycetes, complex metabolic regulation limits productivity and genetic stability, while heterologous hosts face challenges such as improper protein folding and metabolic imbalance (Ameruoso et al., 2022; Ko et al., 2025). Additionally, although structure-guided design enables resistance evasion, predicting in vivo activity and pharmacokinetics remains difficult. Advancing host optimization, metabolic modeling, and computational design frameworks will be essential to fully realize these strategies.

## Conclusion and Future Perspectives

The increasing crisis of AMR has underscored the structural limitations of conventional antibiotic therapies and highlighted the urgent need for next-generation antimicrobial strategies. In this review, we discuss how the integration of synthetic biology approaches into various antibiotic-alternative strategies can address the inherent shortcomings of traditional antibiotics, including their lack of specificity, propensity to induce resistance, and disruption of microbial ecosystems.

CRISPR–Cas systems offer the potential to fundamentally improve the indiscriminate bactericidal effects of conventional antibiotics by selectively eliminating or silencing resistance genes with high genetic specificity. However, the clinical translation of CRISPR-based antimicrobial strategies hinges upon several critical prerequisites, including efficient delivery, overcoming bacterial evasion mechanisms against CRISPR, and the assurance of in vivo safety. Synthetic biology–driven bacteriophage engineering expands the feasibility of precision antimicrobial therapy by overcoming key limitations of conventional phage therapy, such as narrow host range, low infection efficiency, and difficulties in isolation and standardization. Nevertheless, major bottlenecks persist, including the evolutionary adaptability of bacteria, insufficient in vivo stability, and environmental variability within complex microbial ecosystems. Microbiome engineering presents an alternative strategy to suppress the dissemination of resistance genes while preserving microbial community stability through the administration of eLBPs or the direct restructuring of microbial consortia. However, the gastrointestinal environment is far more complex and dynamic than controlled laboratory settings. This creates technical challenges such as limited colonization efficiency of engineered strains, unstable functional expression, and the risk of unintended horizontal gene transfer. In parallel, advances in metabolic engineering and structure-based antibiotic design are reshaping traditional natural product–centered discovery paradigms, enabling the rational development of antimicrobial compounds capable of bypassing known resistance mechanisms. Despite this progress, significant barriers to clinical translation remain, including metabolic imbalance, cytotoxicity, and persistent difficulties in predicting in vivo activity and pharmacokinetic properties. For the practical implementation of these next-generation antimicrobial strategies, it is essential to first establish biosafety assessment guidelines addressing their long-term impacts on human health and the environment, as well as to develop rigorous frameworks capable of validating ecological stability within complex in vivo systems.

The recurring bottlenecks observed across these diverse antimicrobial strategies cannot be fully addressed through the refinement of individual technologies alone, but rather require integrative approaches that emphasize technological convergence. Increasingly, CRISPR-based genetic control systems, synthetic phage engineering, microbiome engineering, and metabolism- and structure-guided antibiotic design are being combined in complementary frameworks to establish a new paradigm for antimicrobial therapy (Ameruoso et al., 2022; Fa-Arun et al., 2023; Kim et al., 2024a). This convergence is further accelerated by AI-enabled design, multi-omics integration, and high-throughput automated platforms supported by biofoundry infrastructures. AI–based models facilitate the prediction of interactions among heterogeneous technological components and enable the rational design of optimal combinatorial strategies (Arnold et al., 2025), while multi-omics data provide a systems-level understanding of the complex interactions among hosts, microbes, and antimicrobial interventions (Ghosh et al., 2025). Furthermore, biofoundry platforms automate the design–build–test–learn (DBTL) cycle, minimizing experimental bottlenecks and substantially enhancing the translational potential of synthetic biology–based antimicrobial strategies (Moffat et al., 2021). Ultimately, such integrative approaches are expected to move the field beyond single-target antimicrobial interventions toward the establishment of precise, sustainable, and evolution-aware therapeutic paradigms for combating AMR.
